# Non-Compaction Ventricle and Associated Cardiovascular and Non-Cardiovascular Diseases; More Attention Is Needed!

**DOI:** 10.3390/life13061231

**Published:** 2023-05-23

**Authors:** Mohammadbagher Sharifkazemi, Reza Mohseni-Badalabadi, Mohammad Kasaie, Leila Ahmadi

**Affiliations:** 1 Cardiology Department, Nemazee Hospital, Shiraz University of Medical Sciences, Shiraz 7193613311, Iran; 2 Tehran Heart Center, Cardiology Department, Tehran University of Medical Sciences, Tehran 5166614711, Iran

**Keywords:** isolated non-compaction of the ventricular myocardium, non-compaction of left ventricular myocardium with congenital heart defects, cardiomyopathy, dilated, with left ventricular non-compaction, aortic aneurysm

## Abstract

Non-compaction of the ventricle (NCV) with a higher tendency to left ventricular involvement (NCLV) is a genetic disorder which can cause arrhythmias and cardiac arrest or remain asymptomatic. It is generally considered an isolated disease most frequently, while a few case reports have reported its association with cardiac anomalies. As the treatment strategies differ for NCV and cardiac anomalies, missed diagnosis of the concomitant cardiac diseases can result in poor response to treatment and prognosis. Here, we present 12 adult patients diagnosed with NCV and associated cardiovascular anomalies. By increasing the clinical suspicion and physician’s awareness about the possibility of the presence of other cardiovascular diseases with NCLV and using close examination and follow-up of the patients, we could diagnose this number of patients during 14 months of investigation. This case series emphasizes the need for increased awareness and attention of echocardiographers on the diagnosis of other cardiovascular diseases associated with NCV for a better response to treatment and improved patient prognosis.

## 1. Introduction

Non-compaction of the ventricle (NCV) with a higher tendency to left ventricular involvement (NCLV) is a rare cardiac disease characterized by a thick bilayered myocardium with trabeculated ventricle and deep intertrabecular recesses. It can be observed as a congenital disease secondary to maturation arrest in the myocardial compaction process during embryogenesis, and also in later life as a gradual and acquired non-embryogenic development of compacted ventricular wall growing into the ventricular lumen in a trabecular fashion [1,2]. A positive family history of cardiomyopathies is observed in 16–34% of patients; most patients are men (62%), and cardiac arrhythmia is reported as the most frequent presentation [3]. However, the patients’ symptoms can vary based on the patient’s phenocopy, ranging from normal left ventricular (LV) systolic function (without clinical manifestations) to dilated cardiomyopathy [4], resulting in life-threatening symptoms such as thromboembolism, heart failure, and sudden cardiac death [5]. Because of the apparent anatomic abnormalities, NCLV is mainly detected as an incidental finding by regular cardiac imaging modalities such as transthoracic echocardiography (TTE) and cardiac magnetic resonance (CMR) imaging [6,7]. Accordingly, some researchers suggest isolated NCLV is not a disease state with no treatments required [8]. However, more attention is required for the non-isolated cases.

As NCLV is a rare cardiac disease and most cases are isolated, few reports are available on the cardiovascular diseases (CVD) associated with NCLV. A review of 35 studies on 2271 patients showed several congenital heart diseases (CHDs) associated in only 7% of cases with NCLV [3] with a variety of cardiac anomalies reported including abnormalities of aortic valves and artery, transposition of the great arteries, ventricular/atrial/atrioventricular septal defect, Ebstein’s malformation, and tetralogy of Fallot [3,9,10]. Here, we present 12 adult patients with cardiovascular anomalies associated with NCLV. We believe that reporting more cases with this condition can help increase the physicians’ and echocardiographers’ attention towards this subject for appropriate diagnosis and treatment of patients with NCLV and associated cardiovascular diseases and thus lead to a better prognosis in such patients.

## 2. Case Presentation

In the present study, we evaluated all patients (N = 1254) who were referred to our centers from April 2021 to July 2022 and were diagnosed with NCLV. The patients were diagnosed with NCLV (by TTE) according to the criteria presented by Jenni and colleagues [11]. The cases were indicated for transesophageal echocardiography (TEE) according to scientific indications and recommended to perform CMR. (NC/C > 2.3 is the generally accepted definition of pathological NCLV with high sensitivity and specificity [12]), as well as genetic tests (N = 1254, by using whole-exome sequencing.) Some patients refused because of economic problems or had medical contra-indications for doing CMR. Moreover, those who had suitable conditions for the necessary echocardiographic views and had a stable cardiac condition (based on electrocardiogram [ECG] results) were indicated for speckle tracking echocardiography (STE) [13]. A summary of the characteristics of the cases is demonstrated in Table 1.

### 2.1. Case #1. NCLV and Coarctation of the Aorta

**Figure 1:**
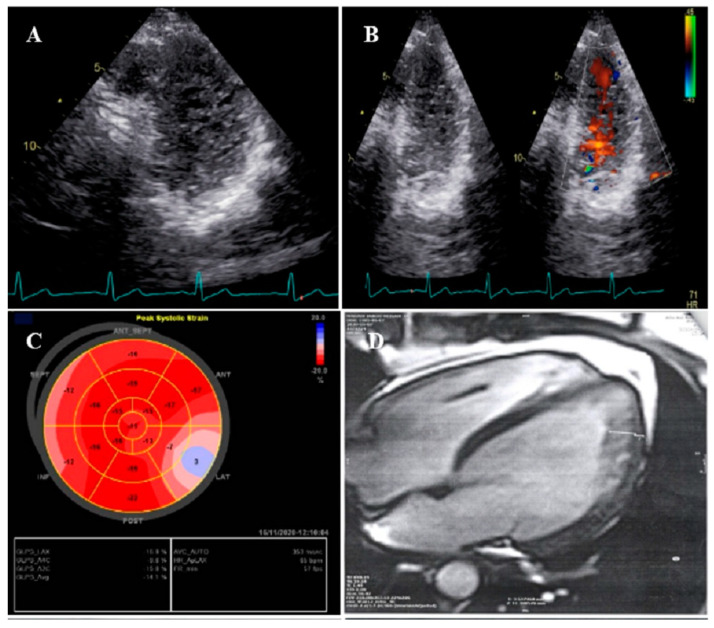

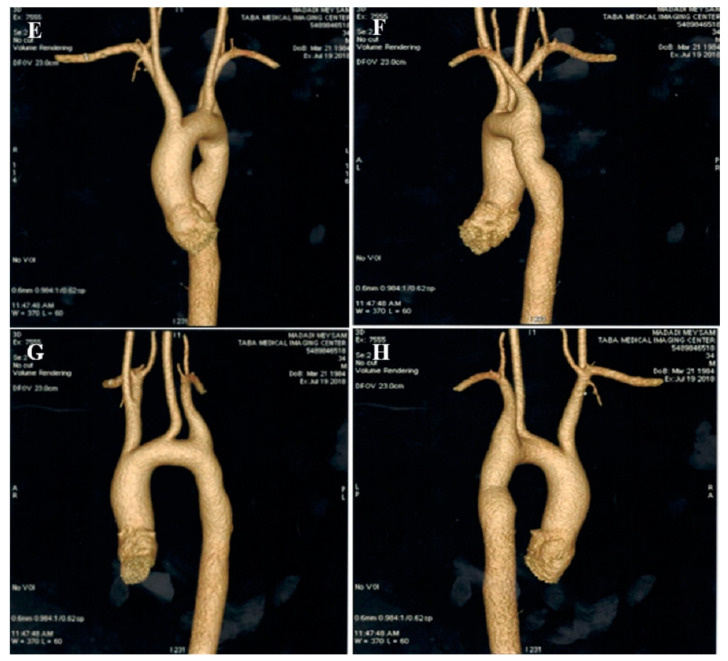
The imaging results of case #1. (**A**); Left ventricular apical short-axis view illustrating hypertrabeculated apical portions in addition to deep intertrabecular recesses, (**B**); Color Doppler echocardiography, showing evidence of direct blood flow from the ventricular cavity into deep intertrabecular recesses (**C**); Speckle tracking echocardiographic findings, compatible with myocardial performance impairment plus relative apical sparing; GLS = −10.4%. (**D**); Prominent trabecular network in the apical lateral segments, (**E**–**H**); Thoracic CT angiography, showing narrowing of descending aorta, distal to left subclavian artery in different projections.

A 47-year-old gentleman who worked in the military service for 15 years was referred to our clinic with a complaint of easy fatigability and mild hypertension since 2 years ago. He was followed up with by another colleague who had the impression of mild LV systolic dysfunction and subaortic web. He received 5 mg daily amlodipine; but had mildly raised blood pressure (BP; 140/90 mmHg). When he was referred to our center, there was a discrepancy between the upper and lower extremities’ BP, and cardiac auscultation revealed a loud A2 closure sound. The review of the previously performed CXR showed posterior rib notching at three points. Computed tomography angiography of the aorta confirmed the presence of post-ductal narrowing in the descending aorta. TTE and TEE showed NCLV with mild LV systolic dysfunction (LVEF = 45%) and coarctation of the aorta with a 28 mmHg rest gradient across the coarctation site. CMR confirmed the diagnosis of NCLV (Figure 1). No other associated pathology was noted. A genetic study showed mutations in the *MYH7* and *MYBPC3* genes; the genetic and echocardiographic results of his siblings (one sister and two brothers) were normal. The patient was recommended to use carvedilol 6.25 mg (three times a day) and spironolactone 25 mg daily. At 1-year follow-up, the patient’s complaints had reduced and the BP was stable.

### 2.2. Case #2. NCLV, Patent Foramen Ovale (PFO), and Fast-Growing Aortic Aneurysm

**Figure 2:**
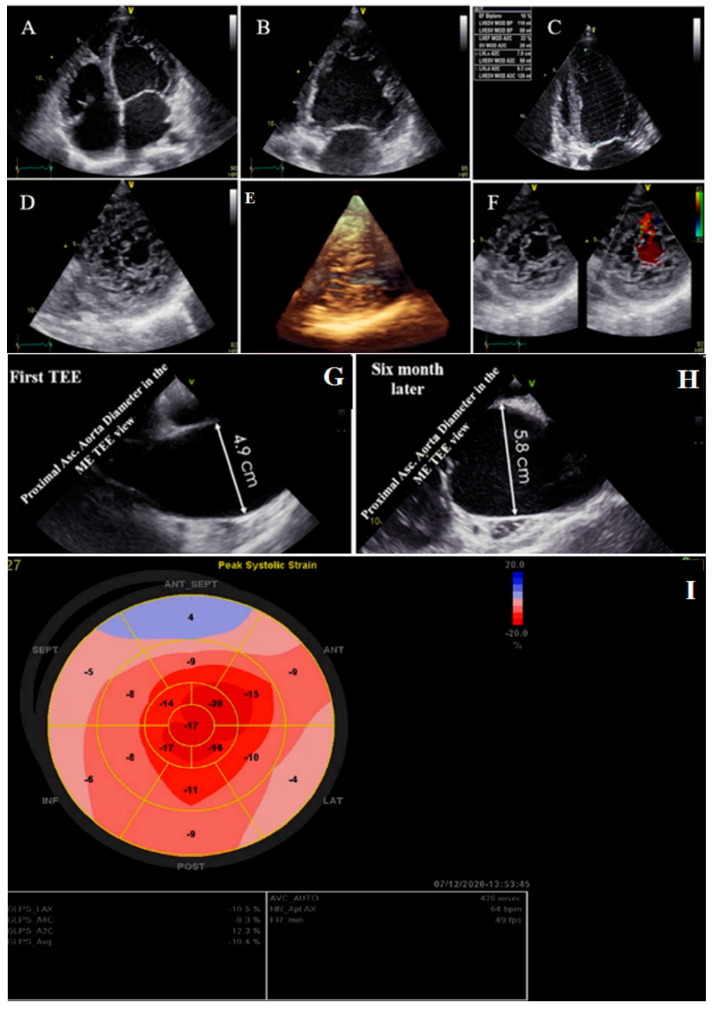
Two- and three-dimensional transthoracic echocardiographic views of case #2: (**A**–**C**); left ventricular apical four and two-chamber views. (**D**,**E**); Apical SAX view, illustrating hypertrabeculated apical portions in addition to deep intertrabecular recesses and reduced left ventricular ejection fraction (LVEF = 16%, calculated by Simpson’s method). (**F**); Color Doppler echocardiography, showing evidence of direct blood flow from the ventricular cavity into deep intertrabecular recesses. (**G**,**H**); Transesophageal echocardiographic findings of baseline (left) and 6 months later (right); follow-up test showed fast-growing aortic root that reached 5.8 cm. (**I**); Speckle tracking echocardiographic findings, compatible with myocardial performance impairment plus relative apical sparing (GLS = −10.4%).

A 56-year-old retired military general was referred with dyspnea on exertion for 3 months, which became worse over time. He had no underlying disease or cardiovascular risk factor. On physical examination, he had tachycardia and tachypnea and used accessory muscles of respiration, and on cardiac auscultation, an audible left-sided S3 gallop with “tic-tac” heart sounds was detected. On ECG, he had sinus tachycardia, low voltage QRS complex, and poor R progression in the precordial leads. TTE showed NCLV with severe global hypokinesia (LVEF = 16%), dilated aortic root and proximal ascending aorta (diameter = 49 mm; indexed = 23 mm), and effacement, in addition to the presence of PFO. A genetic study showed mutations in the *DSP* (e.3857_3859del: p.1286_1287del.), *TTN* (c.C80492T: p. P26831L), and *DSC2* (c.A1886G:p.N629S) genes. A 6-month follow-up TTE showed a fast-growing aortic root aneurysm that reached 58 mm (indexed = 28 mm), although the patient was receiving beta-blockers and had no clinical symptoms. STE showed impaired myocardial performance with relative apical sparing and coronary angiography with the left ventricle and aortic root cineangiography showed dilated LV with remarkable recesses and dilated aortic root (6.25 cm) (Figure 2). The patient was scheduled for a valve-sparing aortic root replacement surgery (David procedure). The perioperative and postoperative TEE showed normal functioning native aortic valve with minimal aortic insufficiency and no aortic stenosis. At the follow-up visit, the patient had no clinical symptoms related to heart failure. The family members refused genetic study because of the high costs of the test (not covered by the insurance).

### 2.3. Case #3. NCLV with Aortic Dilation Complicated by Coronary Embolism

**Figure 3:**
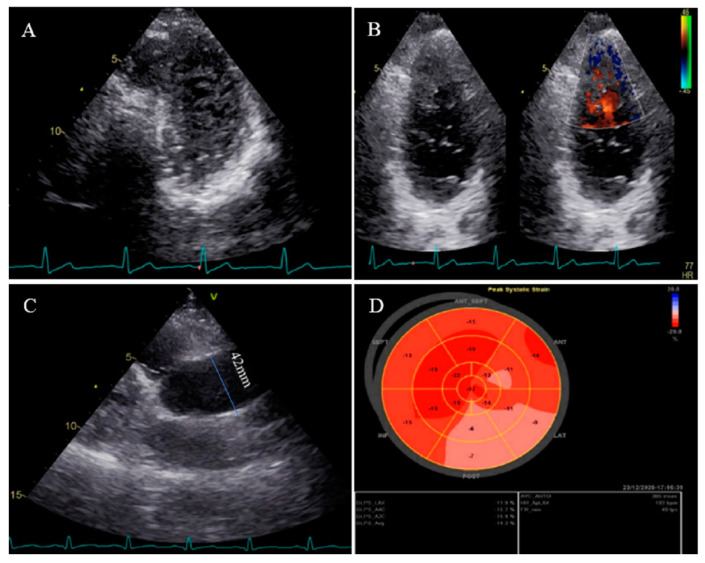

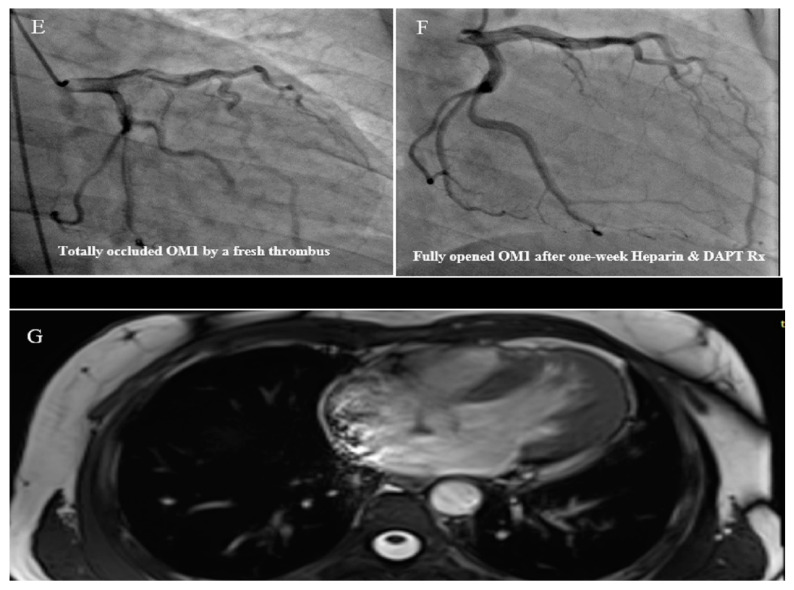
Two-dimensional transthoracic echocardiographic views of case #3. (**A**); Left ventricular apical SAX view, illustrating hypertrabeculated apical portions in addition to deep intertrabecular recesses. (**B**); Color Doppler echocardiography, showing evidence of direct blood flow from the ventricular cavity into deep intertrabecular recesses. (**C**); Dilated aortic proximal ascending aorta. (**D**); Speckle tracking echocardiographic findings, compatible with myocardial performance impairment of all segments with GLS = −14.3%. (**E**,**F**); Coronary angiography showing on arrival and 2-week post-treatment results. (**G**); Cardiac magnetic resonance imaging, illustrating prominent non-compaction in left ventricular apical and lateral wall.

A 37-year-old gentleman with no history of cardiac disease and major CVD risk factors presented with acute retrosternal pain with radiation in both shoulders since 3 h prior to admission to the emergency department, accompanied by profound cold sweating and nausea, though there was no vomiting. On arrival, the ECG showed ST-segment elevation in the inferolateral leads, and diagnostic coronary angiography revealed a filling defect in the second obtuse marginal branch, in favor of occlusion by a fresh thrombus. He spent his hospital course uneventfully with response to anticoagulation plus dual antiplatelet therapy for 2 weeks (opened occlusion on the second angiography) and was discharged after 3 days in stable condition. Because of the possibility of the cardiac source of embolization, TTE and TEE were performed, which revealed NCLV with normal left ventricular ejection fraction (LVEF = 55%) and no obvious thrombus, in addition to mild aortic dilation (ascending aorta diameter = 42 mm; indexed = 22 mm). STE showed reduced global longitudinal strain (GLS = −11%). Moreover, CMR confirmed the diagnosis of NCLV (Figure 3). A genetic study showed two heterozygous mutations in the *SCNIB* and *ALPK3* genes. The family screening was performed, and his mother had an undiagnosed isolated NCLV with the same genetic results, as well. The patient was prescribed warfarin and was symptom free at the follow-up visit.

### 2.4. Case #4. Biventricular Non-Compaction (BVNC) with Ostium Primum Atrial Septal Defect (ASD) Plus Complete Heart Block

**Figure 4:**
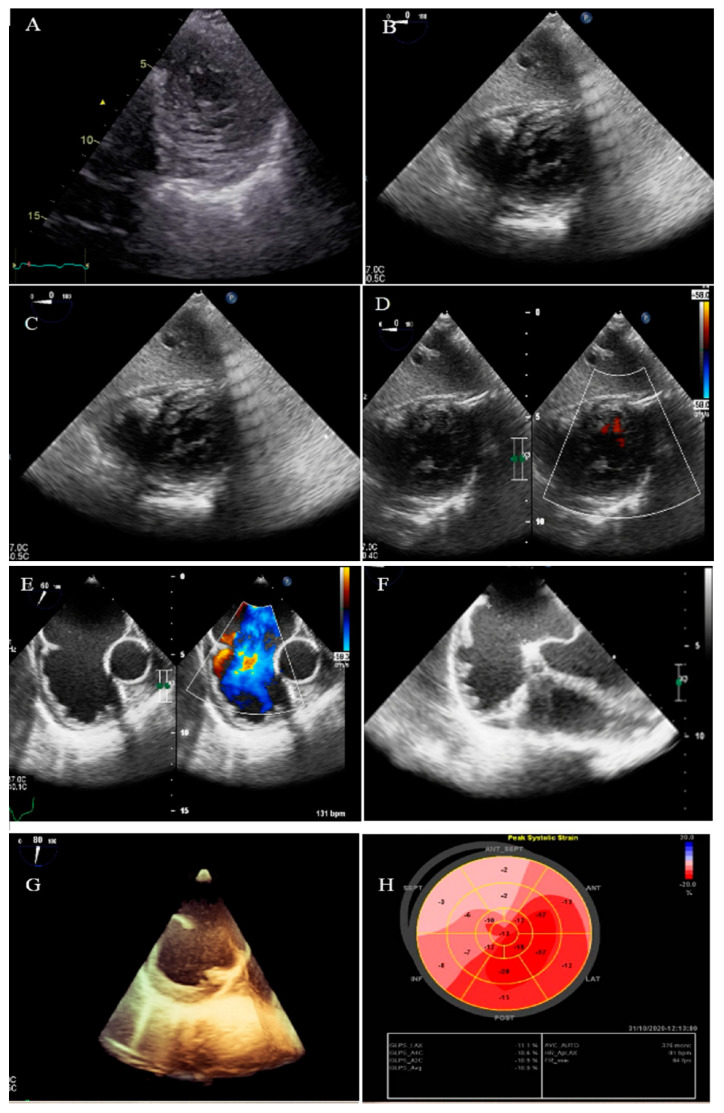

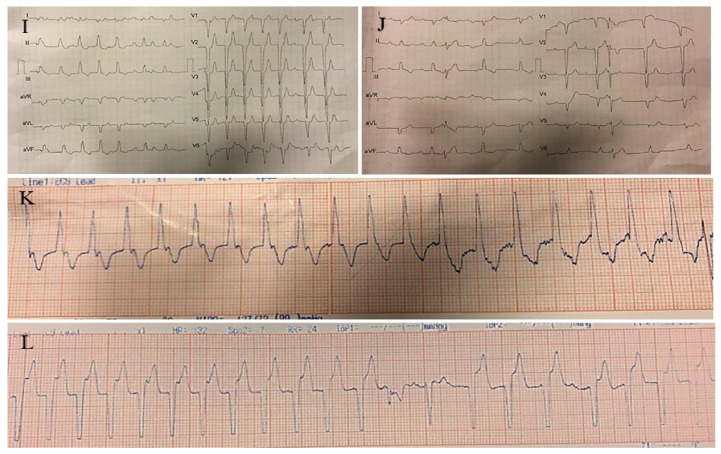
Two-dimensional transthoracic echocardiographic views of case #4, (**A**); Biventricular apical SAX view, (**B**,**C**); Transesophageal gastric views, illustrating hypertrabeculated apical portions in addition to deep intertrabecular recesses. (**D**); Color Doppler echocardiography, showing evidence of direct blood flow from the ventricular cavity into deep intertrabecular recesses. (**E**–**G**); Two- and three-dimensional mid-transesophageal echocardiographic views, showing a large ostium primum type of atrial septal defect with left to right shunt. (**H**); Speckle tracking echocardiographic findings, compatible with myocardial performance impairment, mild in apical to severe in basal segments, and GLS = −10.9%. (**I**–**L**); Twelve-lead electrocardiography and a couple of tracings at different times, showing complete heart block and sustained runs of wide QRS tachycardia.

A 34-year-old lady, 34 weeks pregnant, was referred to the hospital with dyspnea, worsening over the course of the last week. A total of 2 h after admission, she experienced sudden cardiac arrest and was successfully resuscitated. At that time, the ECG monitoring was in favor of torsade de pointes. During the hospital course, intermittent complete heart block was noted. She did not report any CVD symptoms before this. Her first pregnancy was uneventful, and her healthy boy was 3 years old. TTE and TEE revealed non-compaction of the left and right ventricles, mild biventricular systolic dysfunction (global LVEF = 40%, tricuspid annular plane systolic excursion = 14 mm, and RV Sm = 8 mm), a large ostium primum ASD (34 mm), and moderate pulmonary hypertension with the pulmonary flow to systemic flow ratio (QP/QS) of 2.1 (Figure 4). During the 4-week follow-up, she developed a couple of symptomatic ventricular tachycardia, which properly resumed to a normal rhythm by electrical cardioversions. Finally, the heart team decided to schedule her for a cesarean section with temporary pacemaker backup. After the delivery of a living, healthy baby, she refused cardiac surgery. Therefore, based on the patient’s and her family’s request, she underwent implantation of a single chamber implantable cardioverter-defibrillator (ICD). During the 4-month follow-up, it did not record a high ventricular rate.

### 2.5. Case #5. NCLV and Arteria Lusoria

**Figure 5:**
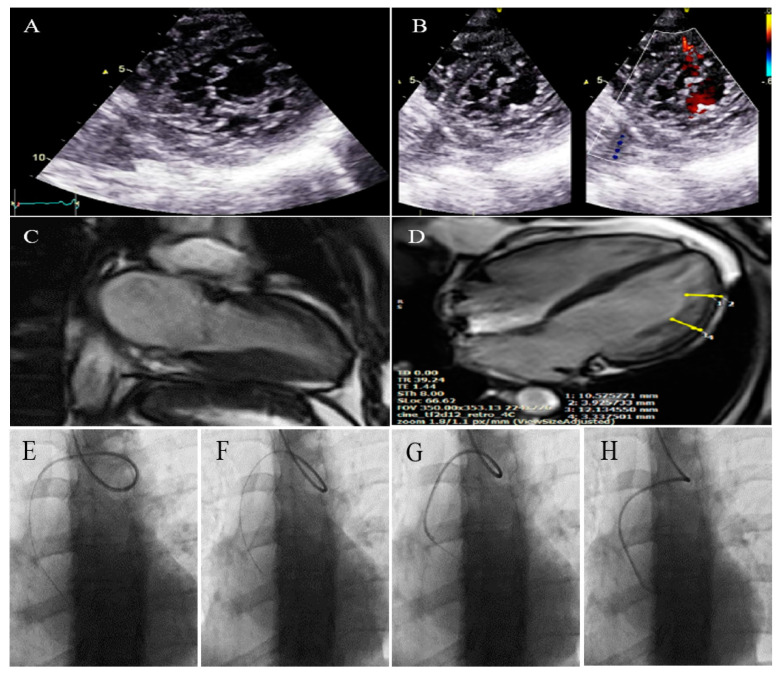
Two-dimensional transthoracic echocardiographic views of case #5. (**A**); Left ventricular apical SAX view illustrating hypertrabeculated apical portions in addition to deep intertrabecular recesses. (**B**); Color Doppler echocardiography, showing evidence of direct blood flow from the ventricular cavity into deep intertrabecular recesses. (**C**,**D**); Cardiac magnetic resonance imaging, illustrating a prominent trabecular network in lateral segments of the left ventricle (LV) and LV apex. The maximum end-diastole NC/C ratio (measured using long-axis images) was 2.7 in the apical lateral segment and 3.6 in the mid-anterolateral segment of LV. (**E**–**H**); Serial images of catheter course during coronary angiography via radial approach, illustrating the arteria lusoria or aberrant right subclavian artery.

A 41-year-old lady was referred to our echocardiographic lab after coronary angiography via radial approach, which revealed an arteria lusoria, a rare congenital arterial anomaly. Coronary angiography had been performed for the strong positive family history of premature coronary artery disease, cardiac symptoms, and equivocal exercise treadmill test result. TTE showed NCLV with preserved systolic function (LVEF = 55%). CMR confirmed the diagnosis of NCLV (Figure 5). A genetic study showed two rare heterozygous mutations in the *IGF2BP2* and *BAG3* genes. The patient did not live in Iran (she was a traveler who came to Iran to meet her family and had no time for further evaluations); therefore, it was recommended that she and her family members follow their heart conditions by referring to a cardiologist.

### 2.6. Case #6. BVNC, Bicuspid Aortic Valve (BAV), and Proximal Muscle Weakness in Lower Extremities

**Figure 6:**
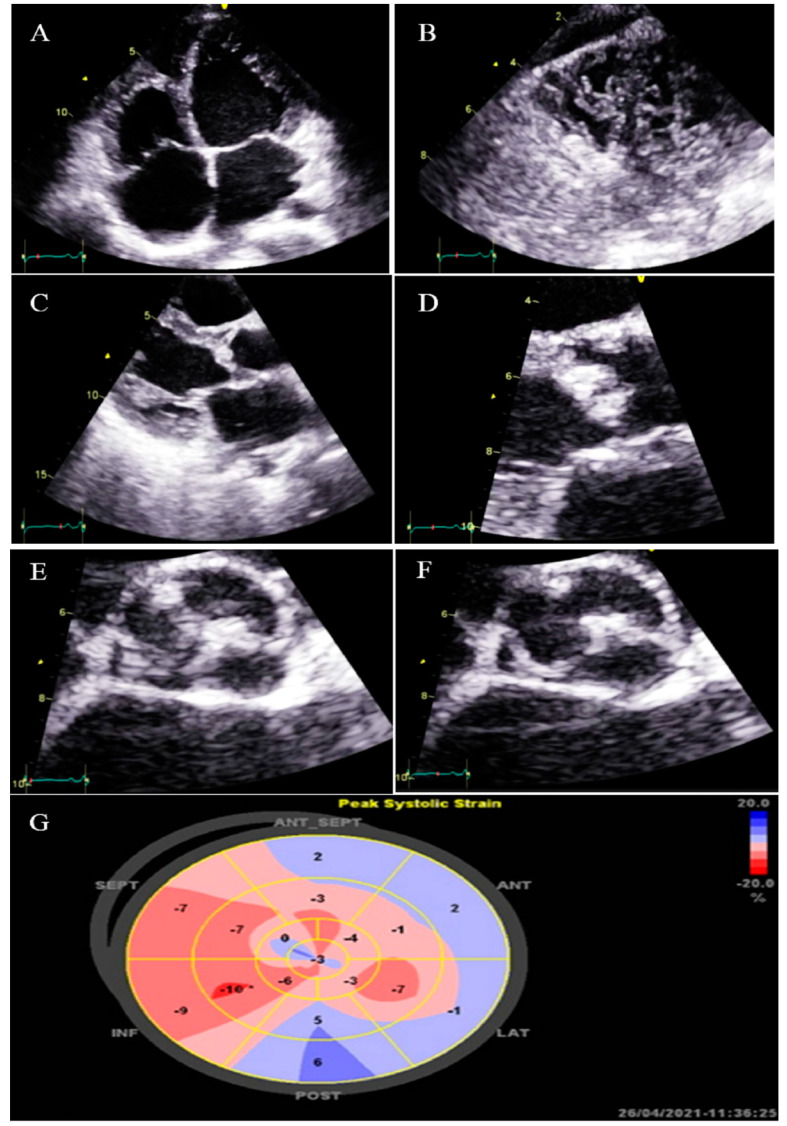

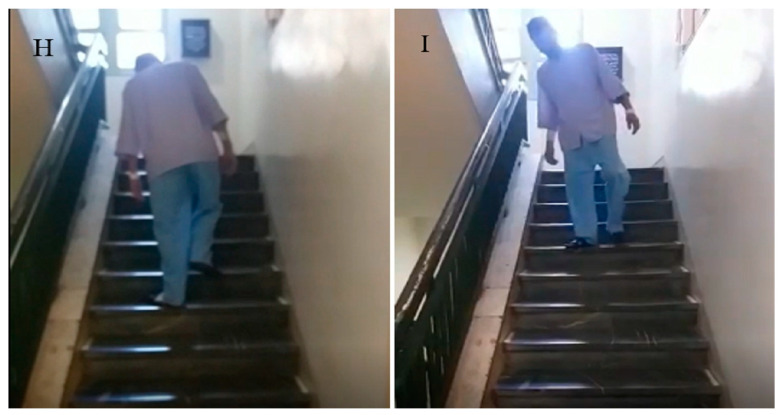
Two-dimensional transthoracic echocardiographic views of case #6. (**A**,**B**); Biventricular non-compaction in apical four-chamber and SAX views. (**C**,**D**); Thick, bicuspid aortic valve with diastolic closure doming; PLAX zoomed-out and zoomed-in views. (**E**); Diastolic closure and systolic opening in SAX view. (**F**,**G**); Speckle tracking echocardiography, illustrating severe myocardial performance impairment in all segments with GLS of −3.3%. (**H**,**I**); Patient’s difficulty in going up and down the stairs because of weakness in the proximal muscles, when he was asked not to use the stair railing.

A 43-year-old gentleman with a complaint of dyspnea on exertion and recently at rest was referred to our hospital. He was labeled as a case of biventricular failure for months by another colleague, who prescribed anti-heart failure medications for him (daily furosemide 40 mg, spironolactone 25 mg, losartan 25 mg, and carvedilol 12.5 mg), medications which have resulted in no marked improvements in his symptoms. He reported generalized weakness and a physical examination revealed proximal muscle weakness in both lower extremities without atrophy. TTE findings were compatible with non-compaction of the left and right ventricles with severe biventricular systolic dysfunction (LVEF = 24% and functional area change = 16%) and BAV (Figure 6). STE showed severe mechanical impairment of myocardial performance in all segments (GLS = −3.3%). He left the hospital because of the lack of insurance coverage and financial problems; therefore, we were unable to evaluate whether his muscle weakness was related to NCLV or not.

### 2.7. Case #7. BVNC, BAV, AS, and Dilated Aorta Ascending Aorta

**Figure 7:**
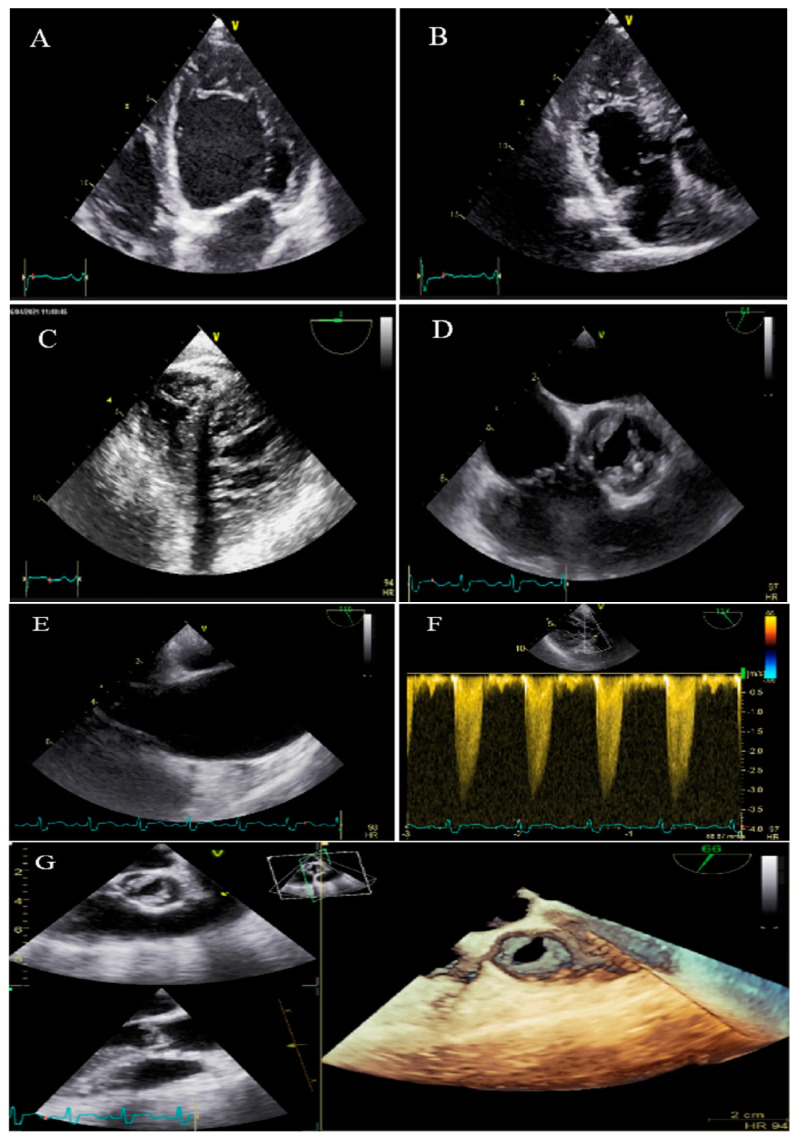
Two-dimensional transthoracic echocardiographic views of case #7. (**A**,**B**); Biventricular non-compaction in apical four and three-chamber views. (**C**–**F**); Transesophageal echocardiography views, illustrating biventricular non-compaction in apical regions in addition to a thick, bicuspid aortic valve with restricted cusps motion (**C**,**D**), dilated ascending aorta (**E**), and increased transvalvular gradient in deep gastric view with a peak velocity of 3 m/s, in favor of moderate aortic stenosis (**F**). (**G**); Full volume three-dimensional transthoracic echocardiography, showing bicuspid, thick, and stenotic aortic valve (AVA by MPR = 1.4 cm^2^).

A 48-year-old gentleman, a medical staffer, was referred to our clinic with dyspnea on moderate exercise and easy fatigability for 6 months, which had become worse over time. On cardiac auscultation, left- and right-sided S4 and S3 gallops, in addition to ejection systolic murmur grade III/VI in the aortic area, were audible. TTE showed non-compaction left and right ventricles with preserved biventricular systolic function, BAV, moderate AS, and dilated ascending aorta (diameter = 47 mm; indexed = 25 mm). At his request, he underwent diagnostic coronary angiography and left/right ventricular cineangiography, and the results showed patent epicardial coronaries plus hypertrabeculation of both ventricles in the biventricular cineangiography (Figure 7). A genetic study showed mutations in the *MYH7* and *MYBPC3* genes. The patient and the family members refused the genetic study because of the high costs of the test (not covered by the insurance). He denied undergoing CMR, due to claustrophobia, and was followed up medically (daily spironolactone 25 mg and carvedilol 6.25 mg twice per day). He was off pills 2 months later and did not return for further follow-up.

### 2.8. Case #8. NCLV with BAV, Dilated Ascending Aorta, and Top Normal Size Main Pulmonary Artery

A 25-year-old lady with a history of palpitation was referred for echocardiography. TTE findings were compatible with NCLV, mildly dilated left ventricle and severe systolic function (LVEF = 32% by Simpson’s), bicuspid aortic valve, and dilated aorta = 44 mm (indexed = 22 mm), and top normal size main pulmonary artery (diameter = 27 mm; indexed = 13.5 mm) (Figure S1). She had been referred to another center to perform CMR but did not return to our clinic.

### 2.9. Case #9. NCLV and BAV

A 37-year-old gentleman was referred to our center for doing echocardiography because of atypical chest pain for a month duration. TTE showed NCLV with normal LV systolic function (LVEF = 55%), plus thick, medial-lateral directed BAV. CMR was requested and was compatible with the diagnosis of NCLV (Figure S2).

### 2.10. Case #10. NCLV with BAV, Highly Redundant and Oscillating Chiari Network

A 48-year-old gentleman was referred to our echocardiography center with a history of worsening palpitation and a suspected right atrial mass on echocardiography which was reported by a cardiologist. TTE in our echo lab showed NCLV, normal LV systolic function (LVEF = 55%), BAV, and a highly redundant and oscillating Chiari network (Figure S3). The patient was referred to the initial center, and we have no information about the results of further evaluations.

### 2.11. Case #11. NCLV, BAV, and Old Myocardial Infarction

A 62-year-old lady was referred to our echocardiography lab for left ventricular systolic assessment before diagnostic angiography. She was a known case of type II diabetes mellitus, hyperlipidemia for years, and ischemic heart disease for 8 months. ECG showed an old inferior wall myocardial infarction. TTE showed NCLV with reduced systolic function (LVEF = 34%, calculated by Simpson’s method), thin and akinetic inferior and posterior wall bases, and BAV (Figure S4). The patient had been a candidate for cardiac surgery with the diagnosis of three-vessel disease, but she left the hospital for personal reasons (discharged against medical advice) and did not refer for a follow-up visit.

### 2.12. Case #12. BVNC with a Dilated Aorta

A 58-year-old gentleman was referred to our clinic with a history of palpitation and shortness of breath on heavy exercise during the past 3 months. TTE showed BVNC with normal systolic function and dilated ascending aorta (diameter = 37 mm; indexed = 22 mm). CMR was compatible with the diagnosis of BVNC (Figure S5). Low-dose carvedilol (6.25 mg three times a day) was started for the patient with the plan of OPD follow-up.

## 3. Discussion

The present case series showed a wide range of cardiovascular anomalies associated with NCV including BAV and other aortic anomalies such as coarctation, dilatation, and aneurysm. A comparison of the diseases reported here with previous studies shows discrepancy among the reported diseases; for instance, the most prevalent diseases (Ebstein’s malformation and tetralogy of Fallot) reported in previous studies [3,9,10] were not observed in our series. According to the wide spectrum of CHDs that can be present simultaneously with NCLV, it is important to pay greater attention to the associated diseases in patients with NCLV, which is not the current state of medicine. Here, we understood that close observation of patients and high clinical suspicion of the echocardiographer could result in the diagnosis of these associations more accurately. We did not perform an epidemiological study and cannot report the prevalence or recommend the most common diseases associated with NCLV, i.e., finding 12 cases in 14 months in a referral echocardiography center, although a high prevalence had been reported in previous reports (12% [10] and 7% [3]).

Previously, NCLV has been considered a disease of children, as the maturation defect during embryogenesis had been suggested as the only mechanism, while later studies suggested that NCLV can be diagnosed in adults as well because of the gradual and acquired non-embryogenic defect [1,2]. The cases presented here also consisted of adults with an average age of 41.4 years (21–62 years), which is close to the mean age reported in a previous review (45.7 years) [3]. Accordingly, it is important that the echocardiographer pays greater attention to the diagnosis of NCLV to prevent the misdiagnosis of patients with idiopathic heart failure or cardiomyopathies [14]. As shown in the present study, two of the patients were previously diagnosed with heart failure and considered as having idiopathic cardiomyopathy, which resulted in a poor response to antifailure medications. This issue is of great significance, as the treatment strategies for NCLV differ from that of idiopathic cardiomyopathy; for example, the indications of anticoagulation therapy are different in patients with NCLV (LVEF < 40%, history of thromboembolism, atrial fibrillation) and should be prescribed sooner than cases with idiopathic cardiomyopathy. Furthermore, patients with NCLV do not benefit much from implantable devices, cardiac resynchronization therapy (CRT), and ICD, which are suggested as an appropriate treatment for idiopathic cardiomyopathy, while patients with NCLV may benefit more from LV remodeling surgery or heart transplantation [15]. Accordingly, it is necessary to have a higher clinical suspicion and train echocardiographers in this regard in order to diagnose NCLV accurately on cardiac imaging.

Another important point emphasized in the present study was attention to the associated cardiovascular diseases, which would have resulted in more complications for the patients if remained undiagnosed. This issue is not only important in patients with decreased LVEF because of the different treatment strategies required, addressed above, but also in patients with normal LVEF (observed in about half of our patients), which may be missed more probably. In some of the cases presented, we observed NCLV in the setting of CHD, implying a hereditary disorder, which may present with or without decreased LVEF; therefore, it is important to differentiate NCLV from cardiac/non-cardiac anomalies, which may occur concurrently in the patient. In the present study, we only included patients who were referred to our center because of their clinical symptoms (dyspnea was the most common presentation) or referred by another cardiologist for echocardiographic assessment. For accurate diagnosis of the associated cardiac and extra-cardiac diseases, we performed additional diagnostic techniques including angiography and STE. Therefore, it is important to increase curiosity and avoid a superficial approach to the patients.

It is also important to pay attention to the differential diagnoses of NCLV, which may include hypertrophic cardiomyopathy (apical type) and dilated cardiomyopathy (that may present as hypertrabeculated apex). In these cases, echocardiographic criteria such as the compaction-to-non-compaction ratio, as mentioned in the present study, should be considered. Further imaging modalities can also help overcome over-diagnosis and prevent false diagnoses. Recent advances in cardiac imaging including CMR, STE, and other non-invasive techniques have enabled a more accurate evaluation of the heart and thus a better investigation in patients suspected of having NCLV [16]. Therefore, it is suggested to perform additional imaging in suspected patients for a more accurate diagnosis of NCLV and the associated diseases. Nowadays, CMR in association with the genetic investigation is considered the gold standard diagnostic method for NCLV and should be therefore applied in all patients with a suspected cardiac abnormality. With this in mind, we could not perform CMR and genetic investigation for all of our patients; some of the patients in our series had financial problems, and others did not accept further investigation; the reason for not performing these investigations has been mentioned in each case.

Considering the hereditary and familial pattern of NCLV [17] and the correlation between genotype and phenotype of NCLV, a genetic study is suggested in all patients to help the physician have a better estimation of patients’ prognosis [18,19]. However, we could only perform whole exome sequencing (WES) analysis in five cases, which showed different genes (*SCNIB*, *ALPK3*, *IGF2BP2*, *BAG3*, *MYH7*, *MYBPC3*, *DSP*, *TTN*, and *DSC2*); a genetic study of the family members also diagnosed cardiomyopathy in the mother of one of the cases. *MYH7*, which has been identified in two cases of our series, has been reported as the most common pathogenic variant [20]. Therefore, genetic mutations are important to be studied for planning the follow-up strategy for the patient.

Furthermore, four cases in our series had simultaneous non-compaction of the right and left ventricles, BVNC. One experienced sudden cardiac arrest 2 h after admission with courses of complete heart block during admission (case #4), and one had BAV and muscle weakness in lower extremities (case #6), both with decreased LVEF. The other had BAV and dilated aorta ascending aorta with normal LVEF, and the last patient had dilated aorta. Other studies have also reported several adverse effects for patients with BVNC such as sudden cardiac death [21,22]. Accordingly, patients with BVNC may even have a worse prognosis compared with those with NCLV, while more studies are required in this regard. Non-compaction of the right ventricle is rarer than the left [23,24,25], and BVNC is considered very rare. However, we assume that finding four cases within 14 months suggests its prevalence is higher than that reported in the literature. Evidence suggests that misdiagnosis is a great concern in patients with BVNC, as the nine patients reported by Wlodarska and colleagues were initially misdiagnosed as arrhythmogenic right ventricular cardiomyopathy [26]. The association of BVNC with Ebstein’s anomaly has also been reported [27,28]. Therefore, more studies are required to determine the pathogenesis, symptoms, genetic mutations, diagnostic methods, and treatment strategies of BVNC, as well as the associated cardiovascular anomalies.

## 4. Conclusions

All in all, the present case series emphasized the considerations required after diagnosis of NCV, which include paying attention to other cardiovascular anomalies associated with NCV and performance of further investigations (including genetic study, CMR, and STE) for estimating the patients’ prognosis and scheduling high-risk patients for close follow-up. The present case series showed that the associated cardiovascular anomalies are not always mild and benign diseases such as PFO, and some may have severe life-threatening diseases such as fast-growing aortic aneurysms, which require quick surgical intervention for saving the patient’s life. Accordingly, it is of great significance to pay attention during echocardiographic assessment to diagnose the associated diseases and select the appropriate treatment to improve the patient’s prognosis.

## Figures and Tables

**Table 1:** The characteristics of the patients presented in the current case series.

N	Sex	Age	Presenting Symptom	CVD History or Risk Factor	Type of Non-Compaction	Associated Cardiovascular Anomalies	Genetic Study	Cardiac Magnetic Resonance Imaging	Treatment	Final Outcome
1	M	47	Easy fatigability and mild hypertension since two years ago	HTN	NCLV, reduced LVEF (45%)	Coarctation of aorta	+	+	Carvedilol 6.25 mg TID plus spironolactone 25 mg daily	Good conditions
2	M	56	Progressive dyspnea	−	NCLV, LVEF = 16%, Global hypokinesia	Hypoplasia of ascending and arch of aorta plus dilated main pulmonary artery	+	−	Scheduled for a valve-sparing aortic root replacement surgery + post-op carvedilol 6.25 mg TID, furosemide 40 mg daily plus spironolactone 25 mg daily	Doing well
3	M	37	Acute retrosternal pain with radiation to both shoulders since three hours prior to admission	−	NCLV, LVEF = 55%	Aortic dilation and coronary embolism	+	+	Anticoagulation plus dual antiplatelet therapy for two weeks switched to lifelong warfarin	Doing well
4	F	34	Dyspnea, two hours after admission, she experienced sudden cardiac death, resuscitated successfully with no sequela	−	BVNC, LVEF = 45%	BAV, ostium primum atrial septal defect plus complete heart block	−	−	Single-chamber implantable cardioverter-defibrillator	No high ventricular rate for 4 months
5	F	41	Echocardiography after angiography	Positive family history for CAD	NCLV, LVEF = 55%	BAV and Arteria Lusoria	+	+	Nil, suggested being under cardiologist follow-up at home country	Did not refer for follow-up
6	M	43	Dyspnea on exertion and recently at rest	−	BVNC LVEF = 24% and fractional area change = 16%	BVNC, BAV, and proximal muscle weakness in lower extremities	−	−	Daily furosemide 40 mg, spironolactone 25 mg, losartan 25 mg and carvedilol 12.5 mg	He left the hospital and did not refer for a follow-up
7	M	48	Dyspnea on moderate exercise and easy fatigability for six months	−	BVNC, LVEF = 55%	BVNC, BAV, aortic stenosis, and dilated ascending aorta	+	Denied because of claustrophobia	Spironolactone 25 mg, carvedilol 12.5 mg daily	Doing well 2 months later. He did not return for a follow-up
8	F	25	History of palpitation referred for the echocardiographic assessment	−	NCLV, LVEF = 32%	BAV, dilated ascending aorta and top normal size main pulmonary artery	−	−	Referred for CMR	Did not refer again to our center
9	M	37	Atypical chest pain for a month	−	NCLV, LVEF = 55%	Medial-lateral directed BAV’s cusps	−	+	Nil	Not yet referred
10	M	48	History of worsening palpitation, and a suspected right atrial mass on echocardiography, reported by a cardiologist	−	NCLV, LVEF = 55%	BAV, highly redundant and oscillating Chiari network	−	−	Suggested referring to his cardiologist in charge for further management	Did not refer again to our center
11	F	62	Echocardiography before diagnostic angiography	Old MI, DM, HLP	NCLV, LVEF = 34%	BAV	−	−	Indicated for CABG according to the result of coronary angiography, she refused and left the hospital	Did not refer again to our center
12	M	58	History of palpitation and shortness of breath on heavy exercise	−	BVNC, LVEF = 55%	Dilated aorta	−	+	carvedilol; 6.25 mg BID	Doing well

Abbreviations: BAV; bicuspid aortic valve, BVNC; biventricular non-compaction, CABG; coronary artery bypass graft surgery, CMR; cardiac magnetic resonance, CVD; cardiovascular disease, DM; diabetes mellitus, HLP; hyperlipidemia, HTN; hypertension, LVEF; left ventricle ejection fraction, MI; myocardial infarction, NCLV; non-compaction of the left ventricle, TID; three times a day, BID; two times a day.

## Data Availability

The raw data supporting the conclusions of this article will be made available by the authors without undue reservation.

## Supplementary Materials

The following supporting information can be downloaded at: https://www.mdpi.com/article/10.3390/life13061231/s1, Figure S1: Two-dimensional transthoracic echocardiographic views of case #8. (A,B); Left ventricular apical four-chamber and apical SAX views, illustrating hypertrabeculated apical portions, in addition to deep intertrabecular recesses and reduced left ventricular ejection fraction (LVEF = 32%, calculated by Simpson’s method). (C); Color Doppler echocardiographic evidence of direct blood flow from the ventricular cavity into deep intertrabecular recesses. (D); PLAX showing dilated ascending aorta and PSAX illustrating bicuspid aortic valve, E; Anteroposterior directed aortic cusps. (F); Top normal size main PA in parasternal RVOT view. (G); Speckle tracking echocardiography, showing significant myocardial performance impairment in all segments with relative apical sparing; GLS= −13.1%; Figure S2: Two-dimensional transthoracic echocardiographic views of case #9. (A); Left ventricular non-compaction in SAX view, in addition to evidence of the direct blood flow from the ventricular cavity into deep intertrabecular recesses on color Doppler echocardiography, (B,C); Thick, medial-lateral directed bicuspid aortic valve. (D,E); Cardiac magnetic resonance imaging, showing prominent trabecular network in lateral segments, as well as left ventricle’s apex, consistent with left ventricular non-compaction; Figure S3: Two-dimensional transthoracic echocardiographic views of case #10. (A,B); Left ventricular non-compaction in A4C and PSAX views as well as redundant and oscillating Chiari network (white arrowhead), (C,D); Thick, unequal aortic cusps size (suggesting bicuspid aortic valve) in PLAX and PSAX views; Figure S4: Two-dimensional transthoracic echocardiographic views of patient #11. (A,B); Dilated left ventricle with apical left ventricular non-compaction in A4C and SAX views, (C); Color Doppler echocardiography, showing direct blood flow from the ventricular cavity into deep intertrabecular recesses, (D,E); PSAX view of the aortic valve, illustrating bicuspid aortic valve with a fusion between non-coronary and right coronary cusps during systole and diastole, respectively; Figure S5: Two-dimensional transthoracic echocardiographic views of patient #12. (A,B); A4C and biventricular apical SAX views, illustrating hypertrabeculated apical portions, in addition to deep intertrabecular recesses, (C); PLAX view, showing dilated ascending aorta (diameter = 37 mm), (D); Color Doppler echocardiography, showing evidence of direct blood flow from the ventricular cavity into deep intertrabecular recesses. (E,F); Cardiac magnetic resonance imaging in different views: SSFP sequence (4-chamber and vertical SA view), displaying the two-layered structure of ventricular myocardium and high signals within trabecular recesses in LV apicoanterior wall and right ventricular apex, suggestive of blood flow communicating with the ventricular cavity, (G); SA view, showing NC/C = 13.4/4.1 = 3.26 in LV apex, (H); SA view, showing NC/C = 18.4/7 = 2.62 in apicoanterior left ventricular wall, and (I); SA view, showing NC/C = 10.6/1.4 = 7.57 in right ventricular apex.
