# Supplementary material for: Non-Compaction Ventricle and Associated Cardiovascular and Non-Cardiovascular Diseases; More Attention Is Needed!
Source: Life (Basel). 2023 May 23;13(6):1231. doi: 10.3390/life13061231 (PMC10303690; doi:10.3390/life13061231)
Supplement: Supplementary file 1 [file life-13-01231-s001.zip › life-2323781-supplementary.pdf]

Supplementary figure legends:

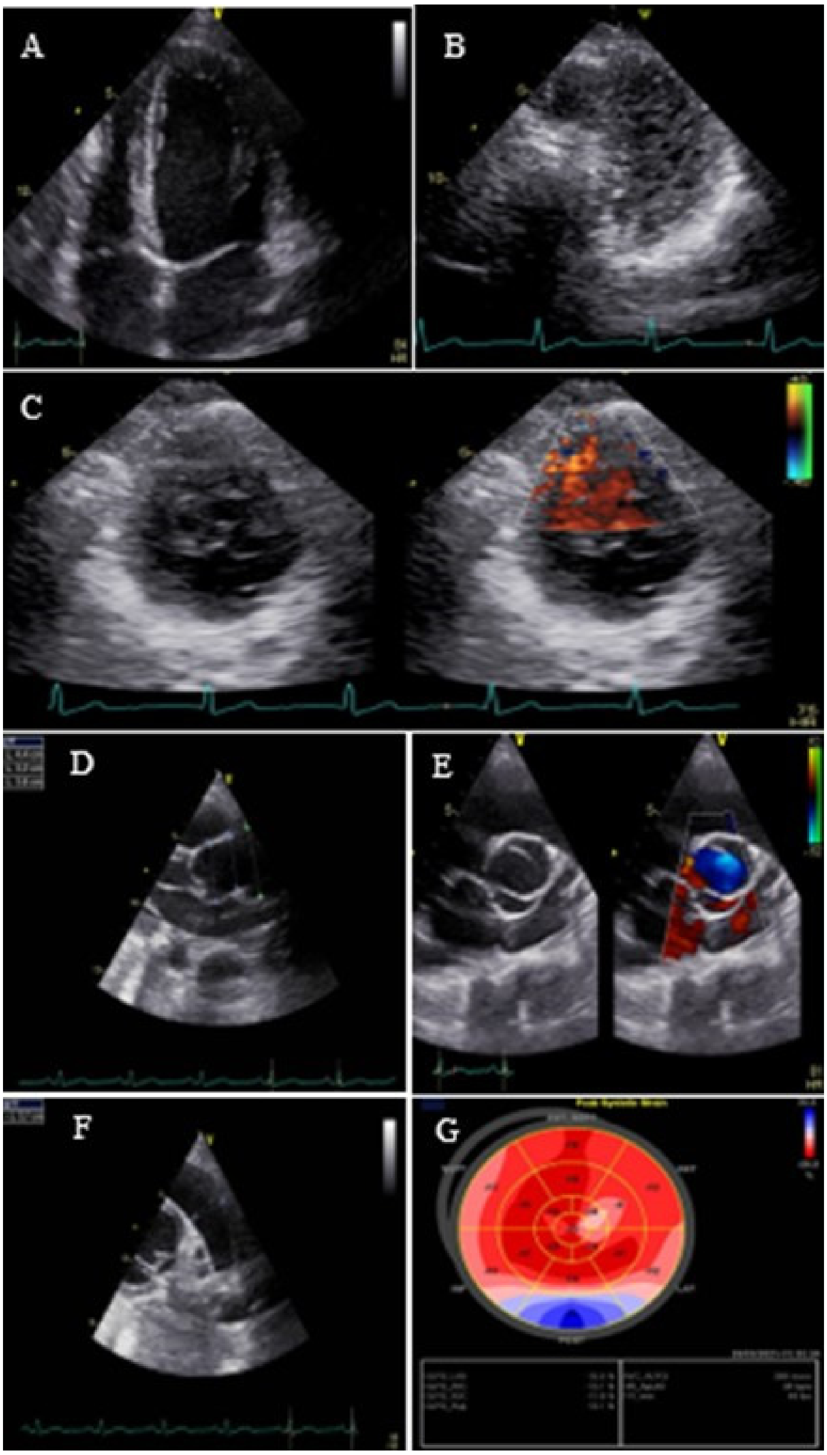

Figure S1. Two-dimensional transthoracic echocardiographic views of case #8. **A and B;** Left ventricular apical four-chamber and apical SAX views, illustrating hypertrabeculated apical portions, in addition to deep intertrabecular recesses and reduced left ventricular ejection fraction (LVEF=32%, calculated by Simpson's method). **C;** Color Doppler echocardiographic evidence of direct blood flow from the ventricular cavity into deep intertrabecular recesses. **D;** PLAX showing dilated ascending aorta and PSAX illustrating bicuspid aortic valve, **E;** Anteroposterior directed aortic cusps. **F;** Top normal size main PA in parasternal RVOT view. **G;** Speckle tracking echocardiography, showing significant myocardial performance impairment in all segments with relative apical sparing; GLS= -13.1%.

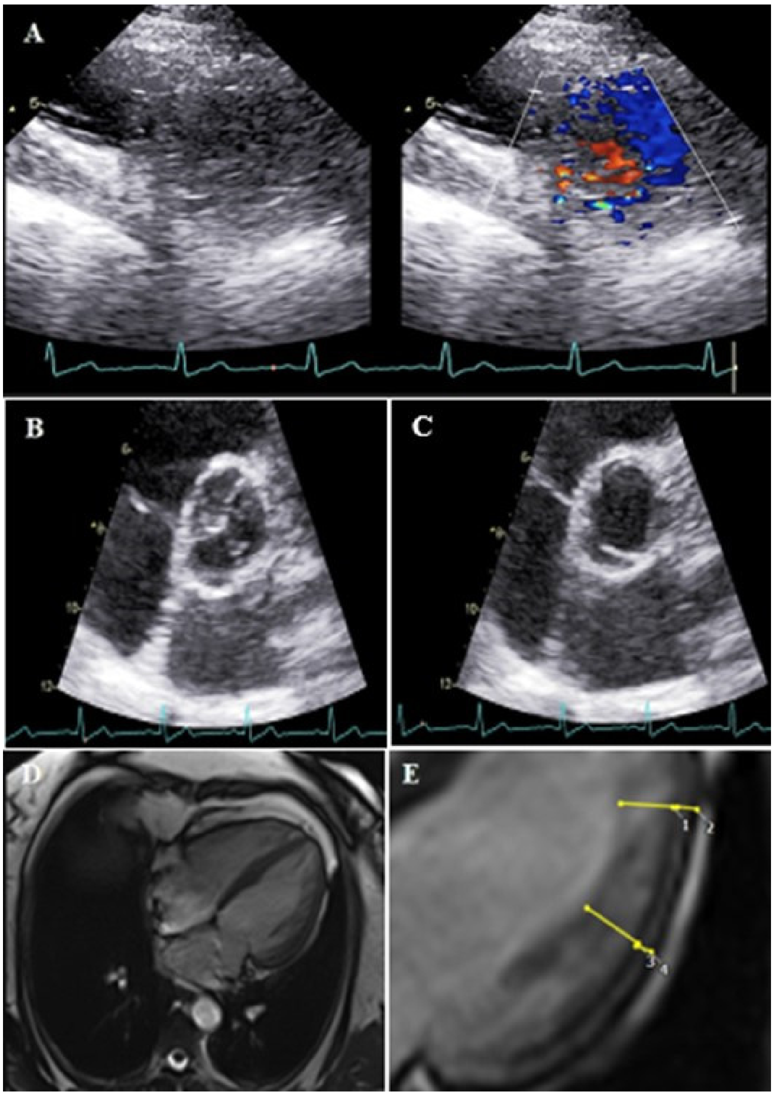

Figure S2. Two-dimensional transthoracic echocardiographic views of case #9. **A;** Left ventricular non-compaction in SAX view, in addition to evidence of the direct blood flow from the ventricular cavity into deep intertrabecular recesses on color Doppler echocardiography, **B and C;** Thick, medial-lateral directed bicuspid aortic valve. **D and E;** Cardiac magnetic resonance imaging, showing prominent trabecular network in lateral segments, as well as left ventricle's apex, consistent with left ventricular non-compaction.

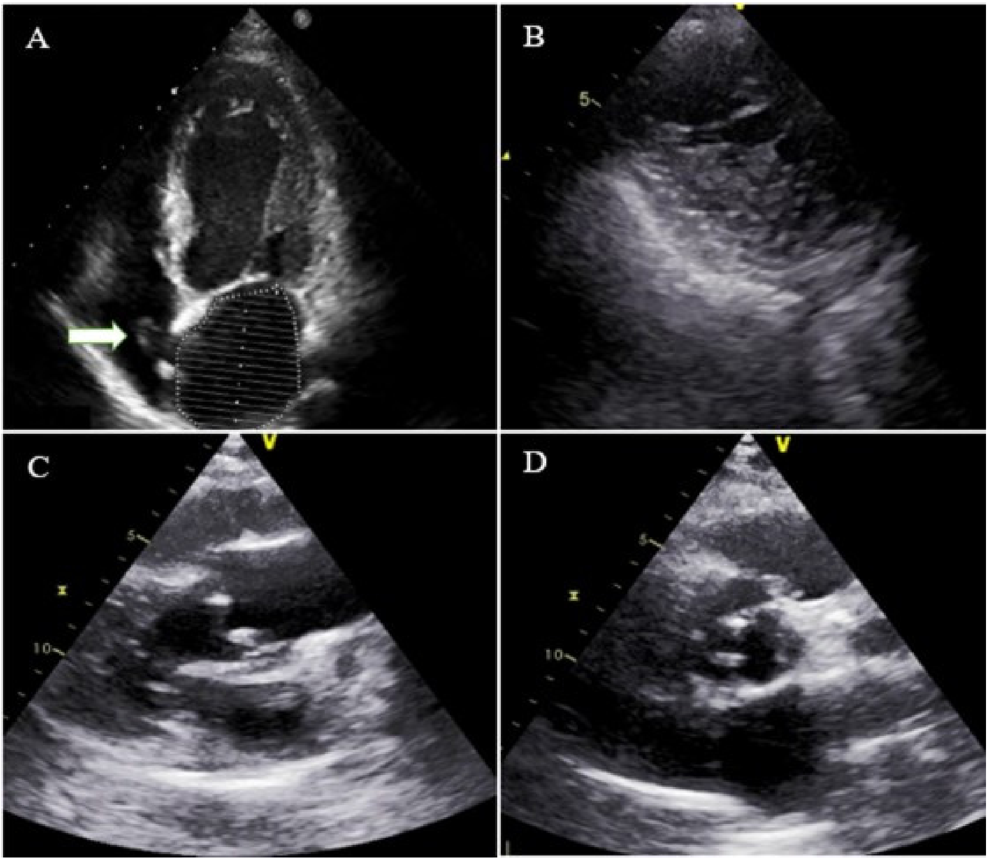

Figure S3. Two-dimensional transthoracic echocardiographic views of case #10. **A and B;** Left ventricular non-compaction in A4C and PSAX views as well as redundant and oscillating Chiari network (white arrowhead), **C and D;** Thick, unequal aortic cusps size (suggesting bicuspid aortic valve) in PLAX and PSAX views.

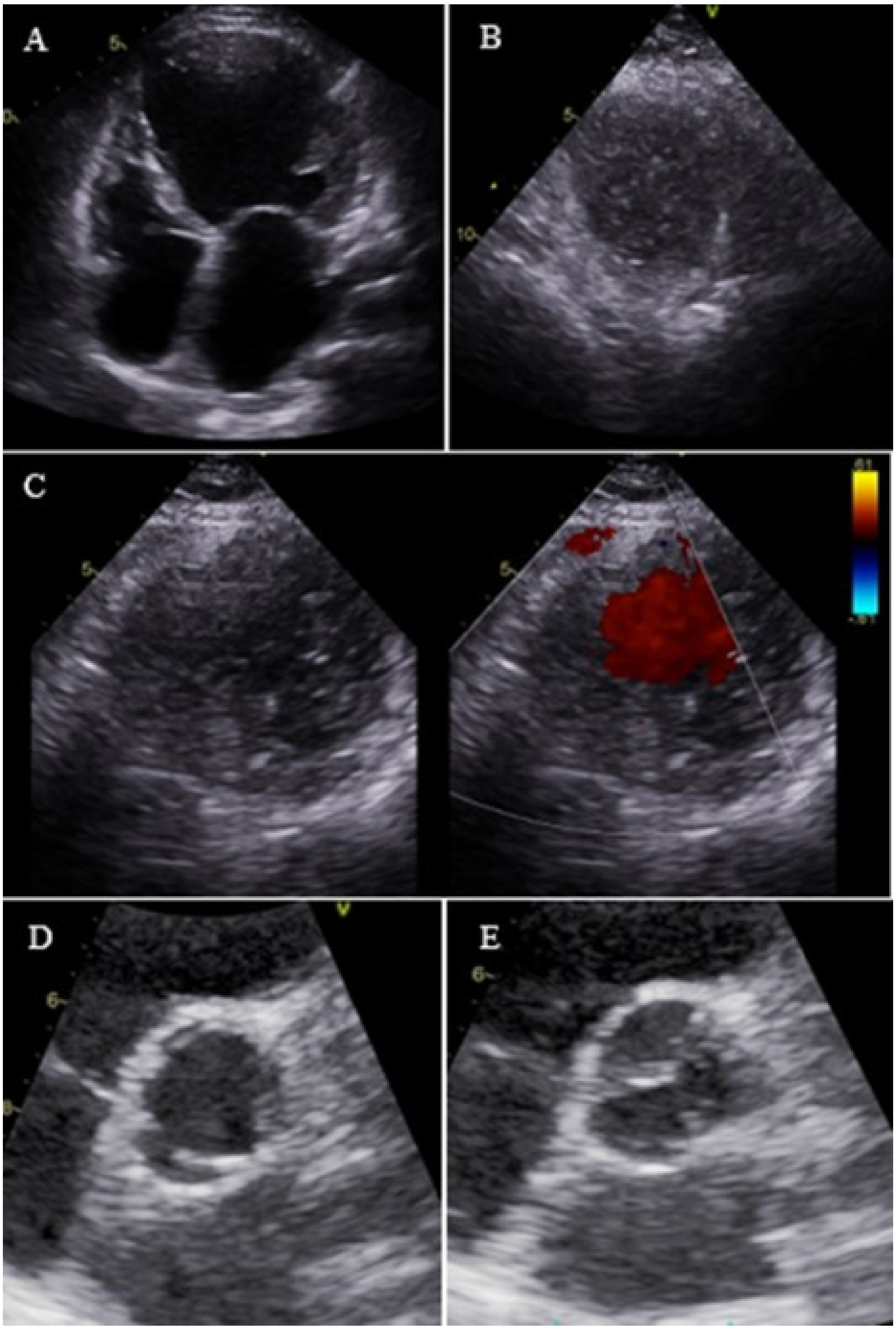

Figure S4. Two-dimensional transthoracic echocardiographic views of patient #11. **A and B;** Dilated left ventricle with apical left ventricular non-compaction in A4C and SAX views, **C;** Color Doppler echocardiography, showing direct blood flow from the ventricular cavity into deep

intertrabecular recesses, **D and E;** PSAX view of the aortic valve, illustrating bicuspid aortic valve with a fusion between non-coronary and right coronary cusps during systole and diastole, respectively.

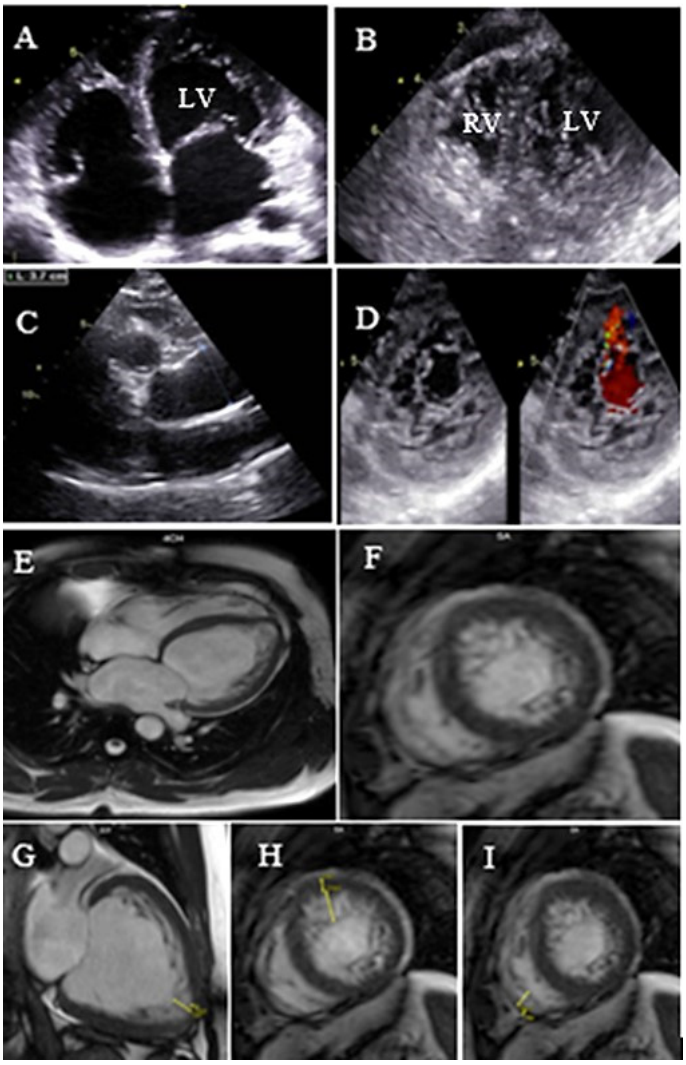

Figure S5. Two-dimensional transthoracic echocardiographic views of patient #12. **A and B;** A4C and biventricular apical SAX views, illustrating hypertrabeculated apical portions, in addition to deep intertrabecular recesses, **C;** PLAX view, showing dilated ascending aorta (diameter=37mm),

D; Color Doppler echocardiography, showing evidence of direct blood flow from the ventricular cavity into deep intertrabecular recesses. **E and F;** Cardiac magnetic resonance imaging in different views: SSFP sequence (4-chamber and vertical SA view), displaying the two-layered structure of ventricular myocardium and high signals within trabecular recesses in LV apicoanterior wall and right ventricular apex, suggestive of blood flow communicating with the ventricular cavity, **G;** SA view, showing NC/C=13.4/4.1= 3.26 in LV apex, **H;** SA view, showing NC/C=18.4/7= 2.62 in apicoanterior left ventricular wall, and **I;** SA view, showing NC/C=10.6/1.4= 7.57 in right ventricular apex.
